# Tumor size and prognosis in patients with Wilms tumor

**DOI:** 10.1016/j.rpped.2014.05.003

**Published:** 2015-03

**Authors:** Valentina Oliveira Provenzi, Rafael Fabiano Machado Rosa, Rosana Cardoso Manique Rosa, Adriana Vial Roehe, Pedro Paulo Albino dos Santos, Fabrízia Rennó Sodero Faulhaber, Ceres Andréia Vieira de Oliveira, Paulo Ricardo Gazzola Zen

**Affiliations:** a Universidade Federal de Ciências da Saúde de Porto Alegre, Porto Alegre, RS, Brazil; b Grupo Hospitalar Conceição, Porto Alegre, RS, Brazil; c Hospital Moinhos de Vento, Porto Alegre, RS, Brazil

**Keywords:** Wilms tumor, Organ size, Drug therapy, Prognosis, Survival analysis

## Abstract

**OBJECTIVE:**

Investigate the relationship of the tumor volume after preoperative chemotherapy (TVAPQ) and before preoperative chemotherapy (TVBPQ) with overall survival at two and at five years, and lifetime.

**METHODS:**

Our sample consisted of consecutive patients evaluated in the period from 1989 to 2009 in an Onco-Hematology Service. Clinical, histological and volumetric data were collected from the medical records. For analysis, chi-square, Kaplan-Meier, log-rank and Cox regression tests were used.

**RESULTS:**

The sample consisted of 32 patients, 53.1% were male with a median age at diagnosis of 43 months. There was a significant association between TVAPQ>500mL and the difference between the TVBPQ and TVAPQ (*p*=0.015) and histologic types of risk (*p*=0.008). It was also verified an association between the difference between the TVBPQ and TVAPQ and the predominant stromal tumor (*p*=0.037). When assessing the TVAPQ of all patients, without a cutoff, there was an association of the variable with lifetime (*p*=0.013), i.e., for each increase of 10mL in TVAPQ there was an average increase of 2% in the risk of death.

**CONCLUSIONS:**

Although our results indicate that the TVAPQ could be considered alone as a predictor of poor prognosis regardless of the cutoff suggested in the literature, more studies are needed to replace the histology and staging by tumor size as best prognostic variable.

## Introduction

Wilms' tumor (WT) accounts for about 6% of all cancers of children and it is the most common malignant renal tumor of childhood. Most are diagnosed before the age of 5, and the current expectation is that over 90% of patients will have an excellent outcome. The majority of children have an asymptomatic and unilateral abdominal mass. Associated symptoms may include hematuria and abdominal pain. This last feature should alert to the risk of tumor rupture, a finding associated with local abdominal recurrence. However, large tumors, usually in advanced stages, did not present indication of surgical intervention and can benefit from preoperative chemotherapy. This may lead to a tumor shrinkage and reduce the chance of complications as tumor rupture.^1^^-^ 3

It is now increasingly important to recognize tumors requiring minimal therapy in order to reduce the burden of treatment and the risk of late effects.^4^ Currently, the most important predictive indicators of recurrence and mortality are staging and tumor histology. The most significant unfavorable factors are high stage and the presence of anaplasia, especially in the diffuse form, which is highly resistant to chemotherapy.^5^^,^ 6 Based on the correlations between the histological features after adjuvant chemotherapy and survival, three prognostic groups of typical renal tumors of childhood were discerned in the *Société Internationale D'oncologie Pédiatrique* (SIOP) studies: low-risk, intermediate-risk and high-risk tumors. This classification is based on the percentage of overall necrosis and the predominant cell type in the residual viable tumor.^7^^,^ 8 The high-risk tumors are associated with poor response to therapy and reduced survival. Furthermore, the influence of tumor response to adjuvant chemotherapy in terms of reducing its volume has been studied, as demonstrated in the studies SIOP 9/German Society of Pediatric Oncology and Hematology (GPOH)^9^^,^ 10 and SIOP 93-01/GPOH.^11^ They raise the possibility that the reduction of tumor volume, besides the classification of histological types of risk, could serve as a new prognostic parameter for the stratification of patients at the time of postoperative treatment. Therefore, currently only GPOH uses tumor volume as a parameter for risk stratification.^12^^,^ 13

The aim of our study was to investigate the relationship of the tumor volume after preoperative chemotherapy (TVAPQ) and before preoperative chemotherapy (TVBPQ) with overall survival at two and at five years, and lifetime.

## Method

Our sample consisted of consecutive patients evaluated in the period from 1989 to 2009 in an Onco-Hematology Service of a reference hospital in southern Brazil. Clinical, histological and volumetric data were collected from the medical records. All patients underwent the SIOP protocol treatment of chemotherapy. This protocol uses neoadjuvant chemotherapy to reduce the tumor volume and the risk of intraoperative rupture.

Tumor volume was calculated according to Weirich et al*,* 9 using the ellipsoid formula: length × depth × thickness × 0.523. The TVBPQ was measured by ultrasound and the TVAPQ was measured in nephrectomy specimen. For analysis, the age of the patients was divided into three groups (0-23 months, 24-47 months and ≥48 months). 

As for staging, the stages I and II were grouped. Histologic types were classified in low, intermediate and high-risk, according to the SIOP 2001 cltassification.^8^ The cases that occurred prior to this publication were classified based on the pathological descriptions. Epithelial and stromal predominant tumors and tumors presenting a predominantly rhabdomyomatous component were also identified. Tumors that either have or have not suffered rupture at time of surgery were also listed. The lifetime was defined as the time from the end of the treatment until the outcome (death or end of study). For the tumor volume, the patients were classified according to the reduction between the TVBPQ and TVAPQ in: (1) poor response (<40%) and (2) good response (≥40%).^10^ We also evaluated whether patients with TVAPQ greater than 500mL had a poorer prognosis, as Reinhard et al^11^ suggested. 

The association between categorical variables was performed using the chi-square test. Survival curves were obtained by Kaplan-Meier and compared by log-rank test. To evaluate the effect of the quantitative parameters, the Cox regression analysis was used. The level of significance was set at 5%, and the analyses were performed using SPSS version 18.0.

## Results

During the assessment period, we identified 32 patients with data available on lesion volume, 53.1% of which were male, with ages at diagnosis ranging from 6 to 87 months (median 43 months). As for the staging, 3.1% were in stage I and V each, and 31.3% in stages II, III and IV each. As for histology, two patients were classified as low-risk (completely necrotic tumor after chemotherapy), 27 as intermediate-risk, and two as high-risk (one with a predominantly blastematous component after chemotherapy and the other with diffuse anaplasia). In one patient it was not possible to apply the classification from the data described in the pathological report. 

Of the patients classified as intermediate risk, three had tumors predominantly epithelial, and three stromal. Only one patient with tumor of intermediate risk showed a predominantly rhabdomyomatous histological type. Only one patient had tumor rupture at the time of surgery. The median TVBPQ was 569.1mL (range 70.6-2,364.2mL) and median TVAPQ was 149mL (range 10-1,468mL). Five patients (15.6%) had TVAPQ>500mL. Of those patients, three were older than 4 years, 3 had stage IV disease, 3 had intermediate-risk histology, and 2 high-risk. Only one of those patients who had TVAPQ>500mL exhibited tumor with stromal predominance. No patient presented a predominantly epithelial tumor or an associated rhabdomyomatous component.

There was a significant association between the TVAPQ>500mL and histologic types of risk (*p*=0.008). No patient with low risk presented TVAPQ>500mL. Three (11.1%) out of 27 patients at intermediate risk and all high-risk patients exhibited TVAPQ>500mL (Table 1). There was also an association between TVAPQ>500mL and the difference between TVBPQ and TVAPQ (*p*=0.015). Nine patients (28.1%) had low response (<40%). Of those, 4 (44.4%) had TVAPQ>500mL. From the 23 patients who showed a good response (71.9%), only one had TVAPQ>500mL (Table 1). 

**Table 1:** Clinical features in patients with Wilms tumor (n=32) according to the TVAPQ (tumor volume after preoperative chemotherapy).

	TVAPQ		p
	<500mL	>500mL	
Age (months)			0.556
0-23	5	1	
24-47	12	1	
≥48	10	3	
Stage			0.301
I-II	9	2	
III-IV	18	3	
Histologic types of risk			0.008
Low	2	—	
Intermediate	25	3	
High	—	2	
Histology			
Epithelial	3	—	0.212
Stromal	2	1	0.142
Rhabdomyomatous	2	2	0.821
Death			
At 2 years	7	2	0.604
At 5 years	8	2	0.584
TOTAL	27	5	

No significant association was found between the difference between TVBPQ and TVAPQ and age of patients (*p*=0.06) and histologic types of risk (*p*=0.092). However, there was an association between the difference between TVBPQ and TVAPQ and predominantly stromal tumors (*p*=0.037) (Table 2). 

**Table 2:** Clinical features verified among the patients with Wilms tumor (n=32) according to the reduction between the tumor volume before preoperative chemotherapy (TVBPQ) and after preoperative chemotherapy (TVAPQ).

Clinical features	Tumor volume reduction (n)		p
	Poor response <40%	Good response ≥40%	
Age (months)			0.06
0-23	4	2	
24-47	2	11	
≥48	3	10	
Stage			0.776
I-II	4	7	
III-IV	5	16	
Histologic types of risk			0.092
Low	1	1	
Intermediate	6	22	
High	2	-	
Histology			
Epithelial	1	2	0.144
Stromal	2	1	0.037
Rhabdomyomatous	2	2	0.659
Lifetime			
At 2 years	3	6	0.685
At 5 years	3	7	0.657
TOTAL	9	23	

**Figure 1:**
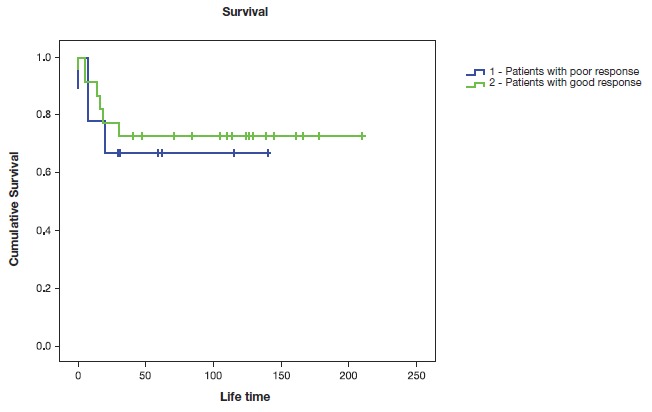
Difference between TVBPQ and TVAPQ and lifetime.

There were nine deaths (28.1%) at two years and 10 (31.3%) at five years. Six of them died due to complications related to the tumor, and four due to occurrence of acute myeloid leukemia (AML) some months after the end of the treatment with chemotherapy. The lifetime ranged from 0 to 210 months. The difference between TVBPQ and TVAPQ was not associated with death at 2 years (*p*=0.685), nor at 5 years (*p*=0.657) (Table 2 and Fig. 1). Also, TVAPQ>500mL was not associated with death at 2 years (*p*=0.604), nor at 5 years (*p*=0.584) (Table 1). However, TVAPQ of all patients was assessed, without a defined cutoff point, and there was an association of this variable with lifetime (HR=1.002, 95%CI: 1.001-1.004, *p*=0.013), i.e., for each increase of 10mL in TVAPQ, there was an average increase of 2% in the risk of death. We did not find association of other variables of the study (age, staging and histology) with the survival.

The difference between TVBPQ and TVAPQ was not associated with tumor stage (*p*=0.776). The difference between TVBPQ and TVAPQ was not associated to predominantly epithelial histology (*p*=0.144), nor to predominantly rhabdomyomatous histological type (*p*=0.659) (Table 2). 

No significant associations were found between TVAPQ>500mL and age (*p*=0.556), stage (*p*=0.301), predominantly epithelial (*p*=0.212) and stromal types (*p*=0.142), and predominantly rhabdomyomatous histology (*p*=0.821) (Table 1). We could not evaluate the influence of tumor rupture over the volume, because it occurred with only one patient. 

## Discussion

The relationship of the TVAPQ and TVBPQ with survival has previously been addressed only by Weirich et al,^10^ Reinhard et al^11^ and Graf et al*.* 14 In our study, the age of the patients was not significantly associated with the difference between TVBPQ and TVAPQ. Nevertheless, it can be observed that 21 (91.3%) out of 23 patients that showed good response to adjuvant treatment were older than 2 years.

The frequency of histological types observed in the present study was similar to that described by Reinhardt et al*.* 11 According to the authors, the TVAPQ is a prognostic factor for intermediate-risk tumors, for which the event-free survival was 70% for those with volumes greater than 500mL, and 93% for those with volumes of less than 500mL. They also concluded that this is especially important for the mixed and regressive histological types (both of intermediate risk), which behave as high-risk tumors. These results are highlighted in the review recently published by Dome et al*.* 13 Despite the small number of patients with low and high-risk histologic types in our study, there was a significant association between TVAPQ>500mL and histologic types of risk. None of the low-risk patients and only 3 (11.1%) with intermediate risk had a TVAPQ>500mL, in contrast to the high-risk patients. This suggests that volumes greater than 500mL tend to fit in cases with high-risk histological tumors, as well as those with volumes of less than 500mL tend to fit in the low-risk cases. 

Conversely, Taran et al^15^ found no statistically significant association between histological risk and tumor volume. However, their study evaluated a smaller number of patients (their sample size was 48 patients) and did not specify the different histologic types of risk.

Although we have not demonstrated an association between histologic types of risk and the difference between TVBPQ and TVAPQ (*p*=0.092), 21 patients at intermediate risk (77.8%) had good response (≥40%) with adjuvant treatment, whereas the patients with high risk had a poor response. The association could be demonstrated with stromal predominant tumors (*p*=0.037). Two of the three cases presenting stromal predominant tumor had poor response to preoperative chemotherapy, which is expected in those cases, as reported by Weirich et al.^9^ However, although these tumors do not respond well to adjuvant treatment, they present a good prognosis, like epithelial predominant tumors.^9^^,^ 16 In the study of Weirich et al,^9^ although there was a poor response to adjuvant chemotherapy in most tumors of epithelial and stromal predominance, none relapsed. According to Verschuur et al,^16^ the median volume reduction after pre-operative chemotherapy was significantly lower for stromal predominant tumors (33%) as compared to other intermediate-risk lesions (67%). As already stated by Beckwith et al,^17^ these features illustrate the independence of aggressiveness and responsiveness in determining the outcome for some patients with cancer. It is worth mentioning that although we did not find a significant association between the predominantly rhabdomyomatous histology and the variables TVAPQ>500mL and the difference between TVBPQ and TVAPQ (probably due to the presence of only one patient with this feature in our sample), previous reports from SIOP trials have shown that this differentiation in the stromal type of WT has a good outcome.^18^

Reinhard et al^11^ found a 5-year event-free survival rate of 91%, but the present study showed a 5-year event-free survival rate of 71%. This difference can be explained by the fact that our sample included patients with metastatic disease (stage IV), while the study of Reinhard et al^11^ included children with localized disease. 

Although we found that patients with tumor volumes greater than 500mL did not show a better response to adjuvant chemotherapy, a significant association between TVAPQ>500mL and prognosis was not observed, unlike Reinhard et al^11^ and Graf et al*.* 14 In the study by Reinhard et al,^11^ patients with TVAPQ greater than 500mL exhibited a worse outcome than those with smaller tumors (70% versus 93% in 5-year event-free survival). Graf et al^14^ evaluated only patients in non-anaplastic stages II and III of WT and verified that time of recurrence and overall survival were univariately and multivariately associated with tumor volume at surgery. 

However, other authors, as Taran et al,^15^ found that tumor volume did not affect survival time. Perhaps our findings are related to the fact that few (five) patients had TVAPQ>500mL. However, without setting a TVAPQ cutoff, every increase of 10mL in volume increased the risk of death in 2%. TVAPQ was the only variable statistically associated to the prognosis. Thus, the use of TVAPQ without a cutoff was the better approach to verify the probability of death in our sample, as already stated by Graf et al*.* 14 Our data, in accordance with Weirich et al,^9,10^ Reinhard et al^11^ and Graf et al,^14^ show that TVAPQ could be used as a contributing factor in the risk stratification of patients submitted to treatment. This aspect is currently being verified in the study protocol SIOP 2001 with intermediate-risk patients.^13^

As showed in the results, the difference between TVBPQ and TVAPQ was not associated to death at 2 and 5 years. Weirich et al^10^ verified that patients who presented a reduction in TVAPQ<40% had a statically significant relapse-free survival (74.3%) and 5-year survival (80.4%).

Four patients of our sample presented and died of AML a few months after the end of treatment. Leukemia has been described as a late effect that may lead to death.^19^ The risk of developing leukemia is higher during the first 5 years following WT diagnosis. Its frequency is highest among those diagnosed after 1990 and it may reflect modifications in the treatment protocols, which decrease the use of radiation therapy and increase the intensity of chemotherapy.^20^

Although our results indicate that the TVAPQ could be considered alone as a predictor of poor prognosis regardless of the cutoff suggested in the literature, more studies are needed to replace the histology and staging by tumor size as best prognostic variables. The histology of the viable tumor remaining after preoperative chemotherapy, whose evaluation was impaired in our study due to the sample size, is still considered the best parameter for risk stratification of patients, together with staging, as showed by Weirich et al,^9^ Reinhard et al^11^ and Graf et al*.* 14 More studies, comprising a larger numbers of patients, are needed to prove these findings.
