# Supplementary material for: Tumor size and prognosis in patients with Wilms tumor
Source: Rev Paul Pediatr. 2015 Mar;33(1):82–7. doi: 10.1016/j.rpped.2014.05.003 (PMC4436960; doi:10.1016/j.rpped.2014.05.003)
Supplement: Translation

# Translation: Tamanho tumoral e prognóstico em pacientes portadores de tumor de Wilms

**Authors:** Valentina Oliveira Provenzi, Rafael Fabiano Machado Rosa, Rosana Cardoso Manique Rosa, Adriana Vial Roehe, Pedro Paulo Albino dos Santos, Fabrízia Rennó Sodero Faulhaber, Ceres Andréia Vieira de Oliveira, Paulo Ricardo Gazzola Zen

**Affiliations:** a Universidade Federal de Ciências da Saúde de Porto Alegre (UFCSPA), Porto Alegre, RS, Brasil; b Grupo Hospitalar Conceição (GHC), Porto Alegre, RS, Brasil; c Hospital Moinhos de Vento (HMV), Porto Alegre, RS, Brasil

**Keywords:** Tumor de Wilms, Tamanho do órgão, Quimioterapia, Prognóstico, Análise de sobrevida

## Abstract

**OBJETIVO:**

Investigar a relação entre o volume do tumor após a quimioterapia pré-operatória (VTPOS) e antes da quimioterapia pré-operatória (VTPRE) com sobrevida geral aos dois e cinco anos e tempo de vida.

**MÉTODOS:**

A amostra foi composta por pacientes consecutivos avaliados de 1989 a 2009, em um serviço de onco-hematologia. Os dados clínicos, histológicos e volumétricos foram coletados a partir dos registros médicos. Para análise, usaram-se os testes qui-quadrado, Kaplan-Meier, *log-rank* e regressão de Cox.

**RESULTADOS:**

A amostra foi composta de 32 pacientes, 53,1% do sexo masculino, com mediana de idade ao diagnóstico de 43 meses. Houve associação significativa entre VTPOS >500 mL e a diferença entre o VTPRE e VTPOS (*p*=0,015) e os tipos histológicos de risco (*p*=0,008). Verificou-se também uma associação entre a diferença entre o VTPRE e VTPOS e o tumor de predomínio estromal (*p*=0,037). Quando se avaliou o VTPOS de todos os pacientes, sem um ponto de corte definido, observou-se associação dessa variável com o tempo de vida (*p*=0,013), isto é, para cada aumento de 10 mL no VTPOS houve um aumento médio de 2% no risco de morte.

**CONCLUSÕES:**

Embora os resultados indiquem que o VTPOS poderia ser considerado um preditor isolado de mau prognóstico, independentemente do ponto de corte sugerido na literatura, mais estudos são necessários para substituir a histologia e estadiamento pelo tamanho do tumor como melhor variável prognóstica.

## Introdução

O tumor de Wilms (TW) é responsável por cerca de 6% de todos os cânceres infantis e é o tumor renal maligno mais comum na infância. A maioria é diagnosticada antes dos cinco anos e a expectativa atual é que mais de 90% dos pacientes terão um excelente desfecho. A maior parte das crianças tem uma massa abdominal assintomática e unilateral. Sintomas associados podem incluir hematúria e dor abdominal. Essa última característica deve alertar para o risco de ruptura do tumor, um achado associado à recidiva abdominal local. No entanto, tumores grandes, geralmente em estágios avançados, não apresentaram indicação de intervenção cirúrgica e podem se beneficiar de quimioterapia pré-operatória. Isso pode levar à redução tumoral e reduzir o risco de complicações como a ruptura do tumor.^1^^,^ 2 ^and^ 3

Presentemente é cada vez mais importante reconhecer tumores que exigem terapia mínima, a fim de reduzir a carga do tratamento e o risco de efeitos tardios.^4^ Atualmente, os indicadores preditivos mais importantes de recorrência e mortalidade são o estadiamento e a histologia do tumor. Os fatores desfavoráveis mais significativos são o estágio avançado e a presença de anaplasia, especialmente na forma difusa, que é altamente resistente à quimioterapia.^5^ ^and^ 6 Com base nas correlações entre as características histológicas após quimioterapia adjuvante e sobrevivência, três grupos prognósticos de tumores renais típicos da infância foram diferenciados nos estudos da Société Internationale D'oncologie Pédiatrique (SIOP): de baixo risco, de risco intermediário e de alto risco. Essa classificação baseia-se na porcentagem de necrose geral e no tipo de célula predominante no tumor residual viável.^7^ ^and^ 8 Os tumores de maior risco estão associados com uma resposta fraca à terapia e sobrevivência reduzida. Além disso, foi estudada a influência da resposta tumoral à quimioterapia adjuvante em termos de redução de seu volume, como demonstrado nos estudos SIOP 9/German Society of Pediatric Oncology and Hematology (GPOH)^9^ ^and^ 10 e SIOP 93-01/GPOH.^11^ Eles levantam a possibilidade de que a redução do volume do tumor, além da classificação dos tipos histológicos de risco, poderia servir como um novo parâmetro prognóstico para a estratificação dos pacientes no momento do tratamento pós-operatório. Portanto, atualmente apenas a GPOH usa o volume do tumor como um parâmetro para a estratificação de risco.^12^ ^and^ 13

O objetivo do nosso estudo foi investigar a relação entre o volume do tumor após a quimioterapia pré-operatória (VTPOS) e antes da quimioterapia pré-operatória (VTPRE) com sobrevida geral aos dois e cinco anos e tempo de vida.

## Método

Nossa amostra foi composta por pacientes consecutivos avaliados de 1989 a 2009 em um serviço de onco-hematologia de um hospital de referência no sul do Brasil. Os dados clínicos, histológicos e volumétricos foram coletados a partir dos prontuários médicos. Todos os pacientes foram submetidos a tratamento quimioterápico com o protocolo da SIOP. Esse protocolo usa quimioterapia neoadjuvante para reduzir o volume do tumor e o risco de ruptura intraoperatória.

O volume do tumor foi calculado de acordo com Weirich et al.,^9^ com a fórmula elipsoide: comprimento x profundidade x espessura x 0,523. O VTPRE foi medido por ultrassonografia e o VTPOS foi medido em amostras da nefrectomia. Para a análise, a idade dos pacientes foi dividida em três grupos (0-23 meses, 24-47 meses e ≥48 meses).

No estadiamento, os estágios I e II foram agrupados. Os tipos histológicos foram classificados em baixo, intermediário e alto risco, de acordo com a classificação SIOP 2001.^8^ Os casos que ocorreram antes dessa publicação foram classificados com base nas descrições patológicas. Também foram identificados tumores predominantemente epiteliais e estromais, que apresentam um componente predominantemente rabdomiomatoso. Os tumores que sofreram ou não ruptura no momento da cirurgia também foram listados. O tempo de vida foi definido como o tempo desde o fim do tratamento até o desfecho (morte ou fim do estudo). Para o volume do tumor, os pacientes foram classificados de acordo com a redução entre o VTPRE e VTPOS em: (1) resposta ruim (<40%) e (2) resposta boa (≥40%).^10^ Também analisamos se os pacientes com VTPOS superior a 500 mL apresentaram pior prognóstico, como Reinhard et al.^11^ sugeriram.

A associação entre as variáveis categóricas foi feita com o teste do qui-quadrado. As curvas de sobrevida foram obtidas pelo método de Kaplan-Meier e comparadas pelo teste de *log-rank*. Para avaliar o efeito dos parâmetros quantitativos, foi usada a análise de regressão de Cox. O nível de significância foi estabelecido em 5% e as análises foram feitas com o programa SPSS, versão 18.0.

## Resultados

Durante o período de avaliação, foram identificados 32 pacientes com dados disponíveis de volume de lesão, dos quais 53,1% eram do sexo masculino, com idades ao diagnóstico de seis a 87 meses (mediana de 43). Quanto ao estadiamento, 3,1% eram estágio I e V cada e 31,3% eram estágios II, III e IV cada. Quanto à histologia, dois pacientes foram classificados como de baixo risco (tumor completamente necrosado após a quimioterapia), 27 como de risco intermediário e dois como de alto risco (um componente predominantemente blastematoso após a quimioterapia e o outro com anaplasia difusa). Não foi possível aplicar a classificação dos dados descritos no relatório patológico em um paciente.

Dos pacientes classificados como de risco intermediário, três tinham tumor predominantemente epitelial e três estromal. Apenas um paciente com tumor de risco intermediário tinha um tipo histológico predominantemente rabdomiomatoso. Apenas um paciente apresentou ruptura do tumor no momento da cirurgia. A mediana do VTPRE foi de 569,1 mL (variação 70,6-2.364,2 mL) e a mediana do VTPOS foi de 149 mL (variação 10-1468 mL). Cinco pacientes (15,6%) tiveram VTPOS > 500 mL. Desses, três tinham mais de quatro anos, três tinham a doença no estágio IV, três tinham histologia de risco intermediário e dois de alto risco. Apenas um desses pacientes que apresentaram VTPOS >500 mL tinha tumor com predominância estromal. Nenhum paciente tinha tumor predominantemente epitelial ou componente rabdomiomatoso associado.

Houve associação significativa entre a VTPOS > 500 mL e os tipos histológicos de risco (*p*=0,008). Nenhum paciente com baixo risco apresentou VTPOS > 500 mL. Três (11,1%) de 27 pacientes de risco intermediário e todos os pacientes de alto risco apresentaram VTPOS > 500 mL (tabela 1). Houve também uma associação entre VTPOS > 500 mL e a diferença entre VTPRE e VTPOS (*p*=0,015). Nove pacientes (28,1%) tiveram baixa resposta (<40%). Desses, quatro (44,4%) tiveram VTPOS >500 mL. Dos 23 pacientes que apresentaram uma boa resposta (71,9%), apenas um apresentou VTPOS > 500 mL (tabela 1).

**Tabela 1:** Características clínicas em pacientes com tumor de Wilms (n = 32) de acordo com o VTPOS (volume do tumor após a quimioterapia pré-operatória)

	VTPOS		*p*
	<500 mL	>500 mL	
*Idade (meses)*			0,556
0-23	5	1	
24-47	12	1	
≥48	10	3	

*Estágio*			0,301
I-II	9	2	
III-IV	18	3	

*Histológico*			0,008
Baixo	2	-	
Intermediário	25	3	
Alto	-	2	

*Histologia*			
Epitelial	3	-	0,212
Estromal	2	1	0,142
Rabdomiomatosa	2	2	0,821

*Morte*			
Em 2 anos	7	2	0,604
Em 5 anos	8	2	0,584
TOTAL	27	5	

Não foi encontrada associação significativa entre a diferença entre VTPRE e VTPOS e a idade dos pacientes (*p*=0,06) e os tipos histológicos de risco (*p*=0,092). No entanto, houve uma associação entre a diferença entre VTPRE e VTPOS e os tumores predominantemente estromais (*p*=0,037) (tabela 2).

**Tabela 2:** Características clínicas verificadas entre os pacientes com tumor de Wilms (n = 32) de acordo com a redução entre o volume do tumor antes da quimioterapia pré-operatória (VTPRE) e após a quimioterapia pré-operatória (VTPOS)

Características clínicas	Redução do volume do tumor (n)		*p*
	Resposta ruim <40%	Resposta boa ≥40%	
*Idade (meses)*			0,060
0-23	4	2	
24-47	2	11	
≥48	3	10	

*Estágio*			0,776
I-II	4	7	
III-IV	5	16	

*Histológico*			0,092
Baixo	1	1	
Intermediário	6	22	
Alto	2	-	

*Histologia*			
Epitelial	1	2	0,144
Estromal	2	1	0,037
Rabdomiomatosa	2	2	0,659

*Tempo de vida*			
Em 2 anos	3	6	0,685
Em 5 anos	3	7	0,657
TOTAL	9	23	

Houve nove mortes (28,1%) em dois anos e 10 (31,3%) em cinco anos. Seis deles morreram devido a complicações relacionadas ao tumor e quatro devido à ocorrência de leucemia mieloide aguda (LMA), alguns meses após o fim do tratamento com quimioterapia. O tempo de vida variou de 0 a 210 meses. A diferença entre VTPRE e VTPOS não foi associada à morte em dois anos (*p*=0,685) nem em cinco anos (*p*=0,657) (tabela 2 e fig. 1). Além disso, o VTPOS > 500 mL não foi associado à morte em dois anos (*p*=0,604) nem em cinco anos (*p*=0,584) (tabela 1). No entanto, o VTPOS de todos os pacientes foi avaliado, sem um ponto de corte definido, e houve uma associação dessa variável com tempo de vida (HR=1,002 IC 95%: 1,001-1,004, *p*=0,013), ou seja, para cada aumento de 10 mL no VTPOS houve um aumento médio de 2% no risco de morte. Não encontramos associação de outras variáveis do estudo (idade, estadiamento e histologia) com a sobrevivência.

**Figura 1:** Associação entre a diferença entre VTPRE e VTPOS e tempo de vida: (1) representa os pacientes classificados como resposta ruim e (2) como resposta boa. Não encontramos associação significativa entre as duas variáveis.

A diferença entre VTPRE e VTPOS não foi associada com o estágio tumoral (*p*=0,776). A diferença entre VTPRE e VTPOS não foi associada à predominância da histologia epitelial (*p*=0,144), nem com o tipo histológico predominantemente rabdomiomatoso (*p*=0,659) (tabela 2).

Não foram encontradas associações significativas entre VTPOS >500 mL e idade (*p*=0,556), estágio (*p*=0,301), tipos predominantemente epiteliais (*p*=0,212) e estromais (*p*=0,142) e histologia predominantemente rabdomiomatosa (*p*=0,821) (tabela 1). Não foi possível avaliar a influência da ruptura do tumor em relação ao volume, pois apenas um paciente apresentou essa ocorrência.

## Discussão

A relação do VTPOS e VTPRE com a sobrevivência foi abordada anteriormente apenas por Weirich et al.,^10^ Reinhard et al.^11^ e Graf et al*.* 14 Em nosso estudo, a idade dos pacientes não foi significativamente associada com a diferença entre VTPRE e VTPOS. No entanto, pôde observar-se que 21 (91,3%) de 23 pacientes que mostraram uma boa resposta ao tratamento adjuvante tinham mais de dois anos.

A frequência dos tipos histológicos observados no presente estudo foi semelhante à descrita por Reinhardt et al*.* 11 De acordo com os autores, o VTPOS é um fator prognóstico para tumores de risco intermediário, para o qual a sobrevida livre de eventos foi de 70% para aqueles com volumes superiores a 500 mL e 93% para aqueles com volumes inferiores a 500 mL. Eles também concluíram que isso é especialmente importante para os tipos histológicos mistos e regressivos (ambos de risco intermediário), que se comportam como tumores de alto risco. Esses resultados são destacados na revisão recentemente publicada por Dome et al*.* 13

Apesar do pequeno número de pacientes com tipos histológicos de baixo e alto risco em nosso estudo, houve uma associação significativa entre VTPOS >500 mL e os tipos histológicos de risco. Nenhum dos pacientes de baixo risco e apenas três (11,1%) com risco intermediário apresentaram um VTPOS > 500 mL, em contraste com os pacientes de alto risco. Isso sugere que os volumes maiores do que 500 mL tendem a se encaixar nos casos de tumores histológicos de alto risco, assim como aqueles com os volumes menores do que 500 mL tendem a se encaixar nos casos de baixo risco.

Diversamente, Taran et al.^15^ não encontraram associação estatisticamente significativa entre o risco histológico e volume do tumor. Entretanto, seu estudo avaliou um número menor de pacientes (o tamanho da amostra foi de 48 pacientes) e não especificou os diferentes tipos histológicos de risco.

Embora não tenhamos demonstrado a associação entre os tipos histológicos de risco e a diferença entre VTPRE e VTPOS (*p*=0,092), 21 pacientes em risco intermediário (77,8%) tiveram boa resposta (≥40%) com tratamento adjuvante, enquanto os pacientes com alto risco tiveram uma resposta ruim. A associação pode ser demonstrada com tumores predominantemente estromais (*p*=0,037). Dois dos três casos que apresentavam tumor predominantemente estromal tiveram resposta ruim ao tratamento quimioterápico pré-operatório, o que é esperado nesses casos, conforme relatado por Weirich et al*.* 9 No entanto, embora esses tumores não respondam bem ao tratamento adjuvante, eles têm um bom prognóstico, como os tumores epiteliais predominantes. 9 ^and^ 16 No estudo de Weirich et al., 9 embora tenha havido uma resposta ruim à quimioterapia adjuvante na maioria dos tumores epiteliais e estromais predominantes, não houve recorrência. De acordo com Verschuur et al., 16 a redução mediana do volume após a quimioterapia pré-operatória foi significativamente menor para tumores predominantemente estromais (33%), em comparação com outras lesões de risco intermediário (67%). Como já foi dito por Beckwith et al., 17 essas características ilustram a independência da agressividade e responsividade na determinação do desfecho para alguns pacientes com câncer. Vale ressaltar que, embora não tenhamos encontrado uma associação significativa entre a histologia predominantemente rabdomiomatosa e as variáveis VTPOS >500 mL e a diferença entre VTPRE e VTPOS (provavelmente devido à presença de apenas um paciente com essa característica em nossa amostra), os relatórios anteriores dos estudos da SIOP mostraram que essa diferenciação no tipo estromal do TW tem um bom resultado. 18

Reinhard et al.^11^ encontraram uma taxa de sobrevivência livre de eventos para cinco anos de 91%, mas o presente estudo mostrou sobrevivência de cinco anos livre de eventos de 71%. Essa diferença pode ser explicada pelo fato de que a nossa amostra incluiu pacientes com doença metastática (estágio IV), enquanto o estudo de Reinhard et al.^11^ incluiu crianças com doença localizada.

Embora tenhamos verificado que os pacientes com volume tumoral superior a 500 mL não mostraram uma melhor resposta à quimioterapia adjuvante, uma associação significativa entre VTPOS > 500 mL e o prognóstico não foi observada, ao contrário de Reinhard et al.^11^ e Graf et al*.* 14 No estudo de Reinhard et al., 11 os pacientes com VTPOS maior do que 500 mL apresentaram um resultado pior do que aqueles com tumores menores (70% versus 93% em sobrevida de cinco anos livre de eventos). Graf et al. 14 avaliaram apenas pacientes em estágios não anaplásicos II e III de TW e verificaram que os tempos para recorrência e sobrevida global foram univariada e multivariadamente associados com o volume do tumor no momento da cirurgia.

No entanto, outros autores, como Taran et al.,^15^ constataram que o volume do tumor não afetou o tempo de sobrevivência. Talvez nossos resultados estejam relacionados ao fato de que poucos (cinco) pacientes apresentaram VTPOS >500 mL. No entanto, sem definir um ponto de corte para o VTPOS, cada aumento de 10 mL de volume aumentou o risco de morte em 2%. O VTPOS foi a única variável estatisticamente associada ao prognóstico. Assim, o uso do VTPOS sem ponto de corte foi a melhor abordagem para verificar a probabilidade de morte em nossa amostra, como já foi dito por Graf et al*.* 14 Nossos dados, em acordo com Weirich et al., 9 ^and^ 10 Reinhard et al. 11 e Graf et al., 14 mostram que o VTPOS pode ser usado como um fator que contribui para a estratificação de risco de pacientes submetidos a tratamento. Esse aspecto está sendo atualmente verificado no protocolo de estudo da SIOP 2001 com pacientes de risco intermediário. 13

Como mostrado nos resultados, a diferença entre VTPRE e VTPOS não foi associada à morte em dois e cinco anos. Weirich et al.^10^ verificaram que pacientes que apresentaram uma redução no VTPOS <40% tiveram uma sobrevida livre de recidiva (74,3%) e uma sobrevida de cinco anos (80,4%) estatisticamente significativas.

Quatro pacientes da nossa amostra apresentaram e morreram devido à LMA alguns meses após o fim do tratamento. A leucemia tem sido descrita como um efeito tardio que pode conduzir à morte.^19^ O risco de desenvolvimento da leucemia é mais elevado durante os primeiros cinco anos após o diagnóstico do TW. Sua frequência é maior entre os pacientes diagnosticados depois de 1990 e pode refletir modificações nos protocolos de tratamento que diminuem o uso de terapia de radiação e aumentam a intensidade da quimioterapia.^20^

Embora nossos resultados indiquem que o VTPOS poderia ser considerado isoladamente como um preditor de mau prognóstico, independentemente do ponto de corte sugerido na literatura, mais estudos são necessários para substituir a histologia e o estadiamento pelo tamanho do tumor como melhores variáveis de prognóstico. A histologia do tumor viável remanescente após a quimioterapia pré-operatória, cuja avaliação foi prejudicada em nosso estudo devido ao tamanho da amostra, ainda é considerada o melhor parâmetro para a estratificação de risco dos pacientes em conjunto com o estadiamento, como mostram Weirich et al.,^9^ Reinhard et al.^11^ e Graf et al*.* 14 Mais estudos, incluindo um maior número de pacientes, são necessários para comprovar esses achados.
